# Evolution of Centrality Measurements for the Detection of Essential Proteins in Biological Networks

**DOI:** 10.3389/fphys.2016.00375

**Published:** 2016-08-26

**Authors:** Mahdi Jalili, Ali Salehzadeh-Yazdi, Shailendra Gupta, Olaf Wolkenhauer, Marjan Yaghmaie, Osbaldo Resendis-Antonio, Kamran Alimoghaddam

**Affiliations:** ^1^Hematology, Oncology and Stem Cell Transplantation Research Center, Tehran University of Medical SciencesTehran, Iran; ^2^Department of Systems Biology and Bioinformatics, University of RostockRostock, Germany; ^3^CSIR-Indian Institute of Toxicology ResearchLucknow, India; ^4^Human Systems Biology Laboratory, RAI-UNAM and INMEGENMexico City, Mexico

**Keywords:** essentiality, centrality, biological network, topological network analysis, biological centrality

## Introduction

Current breakthroughs in high-throughput technologies have propelled the development of databases that systematically store knowledge of how genes, proteins, and metabolites interact. To elucidate the mechanisms of molecular interaction, such data can be represented through networks where nodes are biological entities (e.g., gene, protein, miRNA, transcription factor, and metabolites) and edges are associations/interactions between them (e.g., co-expression, signaling, regulation, and physical interaction). One approach to use such networks is to analyze their topological structure and try to relate this to biological function.

Topological analysis hints at the possible behavior of a network in the regulation of biological processes or phenotypes and help in unveiling the core mechanisms. Broadly speaking, topological parameters can be used to explore: (1) collective behaviors (global properties such as diameter, small-world and scale-free properties of a network), (2) subnetwork behaviors (functional motif discovery), and (3) individual behaviors (prioritization of important nodes by centrality indices) of various network components (Ma and Gao, 2012).

One of the first attempts found in the literature considered centrality related to lethality, and is known as the centrality–lethality rule proposed by Jeong et al. indicating a positive correlation between connectivity and indispensability in the yeast protein-protein interaction map (Jeong et al., 2001). Similarly, Wagner and Fell analyzed the structure of a large metabolic network of *E. coli* using metabolite node degree and shortest mean path length and observed small world like properties that follow power-law distributions (Wagner and Fell, 2001). In these two comprehensive studies, an old metric system (centrality index) was applied with different strategies, aiming to answer the following question: Do centrality indices predict the essential nodes in the biological networks?

Remarkably, topological analyses carried out in transcriptional regulatory (TR) and metabolic networks have been a valuable guide to identify those biological components, called essential nodes, that play a major role in vital functional activities for some microorganisms (Resendis-Antonio et al., 2005, 2012). The relationship between nodes topological features, such as their degree, and their essentiality remains however debated (Coulomb et al., 2005).

Prediction of essential proteins is a challenging task because it needs experimental approaches that are expensive, time-consuming, and laborious (Zhong et al., 2013; Li et al., 2014). To optimize the search of essential nodes in biological networks, a series of computational methods that include topological criteria have been proposed. In this paper, we review the cutting edge computational methods by categorizing them according to their underlying strategies to identify essential components. In each case, we discuss their predictive experimental power and identify shortcomings.

## First strategy: use of individual classical centrality index

The most commonly used centrality index is the degree centrality which is calculated as the number of direct connections to a node. Many studies suggested that highly connected nodes or “hubs” are more likely to be essential (Hahn and Kern, 2005; Joyce and Palsson, 2008). For instance, in 2005, Hann and Kern compared centrality and essentiality in yeast, worm and fly PPI networks and concluded that a protein connectivity has an effect on the probability of being essential (Hahn and Kern, 2005). Nevertheless, high connectivity does not necessarily imply its essentiality. In 2005, Mahadevan and Palsson, indicated that in genome-scale metabolic models of *E. coli, S. cerevisiae*, and *Geobacter sulfurreducens*, the essentiality is not correlated with node connectivity (Mahadevan and Palsson, 2005). In addition, in 2007, Tew et al. concluded that in the PPI network a low-connectivity node could also be considered as essential (Tew et al., 2007). To improve upon this, other metrics were suggested to predict essential genes. Thus, almost all classic centrality indices (Freeman, 1979) that were developed for characterizing social networks (such as the degree, closeness, and betweenness centralities) were applied to biological networks. For instance, in 2004, Koschützki and Schreiber applied five centrality indices (degree, eccentricity, closeness, random walk betweenness, and Bonacich's eigenvector) to the PPI network of *Homo sapiens* and gene regulatory network of *E. coli*. They showed that eccentricity and eigenvector are highly correlated in the PPI network while within the TR network a strong correlation between eigenvector and closeness was observed (Koschützki and Schreiber, 2004). Betweenness centrality is based upon the frequency with which a node lies between the shortest communication path of all other possible pairs of nodes within a network and highlights the gatekeepers of communication within the network. Eccentricity centrality of a node is calculated as the reciprocal of the maximum of shortest path lengths from that node to all other nodes in the network. Thus, the node with highest eccentric centrality is considered as the most central node in a network. In contrast the closeness centrality is measured by the reciprocal of sum of the geodesic distances from that node to all other nodes in the network. The basic idea behind the eigenvector centrality of a node was the assumption that centrality index of a node is not only determined by its position in the network but also by the neighboring nodes. Overall degree, betweenness and closeness centrality measurements were among the most common topological parameters investigated in terms of biological network analyses. Potapov et al. introduced a new centrality measurement, named pairwise disconnectivity index, to qualify the importance of individual nodes and/or interactions for sustaining the communications between connected pairs of nodes in a directed network (Potapov et al., 2008). The authors discussed some of the limitations of the betweenness centrality index, mainly the identification of the shortest path for the communication between a pair of nodes. They argued that the importance of a path does not depend on the length but on other factors, such as the concentration of the species, rate constant etc. Thus, even the longer path can be faster and efficient in biological scenarios. Moreover, the peripheral nodes were not considered. However, in 2014, Raman et al. analyzed the PPI network of a diverse set of 20 organisms. They computed parameters such as degree, betweenness, closeness, and pairwise disconnectivity indices and demonstrated that degree and betweenness centralities correlate with lethality in many organisms but closeness and pairwise disconnectivity indices are not strong indicators of essentiality (Raman et al., 2014).

## Second strategy: combination of classical centrality measures

Some researchers have also attempted to combine the individual centrality matrices to achieve more accurate results. They believe that a single measure of centrality does not solely predict the essential nodes in biological networks. Therefore, combining different centrality indices could yield better results. Examples of such studies include the work of Gabriel del Rio et al. in 2009 on the prediction of essential genes using a new score based on the combination of two or more existing centrality indices (del Rio et al., 2009). They analyzed 16 different centrality measures on 18 reconstructed metabolic networks for *S. cerevisiae* and explained that no single centrality measure identifies essential genes while the combination of at least two centrality measures achieves a reliable prediction. More specifically, they observed that combining “1/clustering coefficient” with either closeness, excentricity, 1/excentricity or radiality resulted in significant prediction of essential genes while no improvement was achieved when three or four centrality measures were combined together (del Rio et al., 2009). Wang et al. performed principal component analysis (PCA) to combine eight centralities, and generated a new integrative node importance measure, structurally dominant proteins index, to find more important nodes in the PPI networks. The proposed integrative measure is strongly correlated with eigenvector, semi-local, network motif, degree, and betweenness measures (Wang et al., 2014). The most recent study, named composite centrality, offered a unified scale to measure node, and edge centralities for general weighted and direct complex evolving networks (Joseph and Chen, 2014).

## Third strategy: use of novel centrality concepts

In addition to the use of individual classical centrality measures and their combinations to identify essential/lethal nodes in biological networks, new indices were designed using other features associated with nodes in biological networks. For instance, Yu et al. in 2004 introduced the notion of marginal essentiality which states that the essentiality of a gene is directly associated to its connectivity and the number of functions of that gene (Yu et al., 2004). Estrada and Rodriguez-Velazquez, in 2005 proposed a new index, subgraph centrality (SC) which characterizes the contribution of each node in all subgraphs of a network. The authors claimed that SC index is better in discriminating the nodes of a network than alternate classical measures such as degree, closeness, betweenness, and eigenvector centralities and is more highly correlated with the lethality of individual proteins removed from the proteome (Estrada and Rodriguez-Velazquez, 2005). Tew et al. defined a functional centrality as the topological centrality within a subnetwork of proteins with similar functions, called neighborhood functional centrality (NFC). NFC predicted the lethal proteins in four *S. cerevisiae* PPI datasets and was able to detect low connectivity lethal proteins that were previously undetected by conventional methods (Tew et al., 2007). Then, Koschutzki and Schreiber demonstrated that motif-based centralities yield better results in gene regulatory networks (Koschützki and Schreiber, 2008). Efforts were made to better predict and improve the existing methods for new insights of centrality usage in biology. For example, Hart et al. used an unsupervised probabilistic scoring scheme on large-scale yeast mass-spectrometry data, emphasizing that essentiality is the product of protein complexes rather than individual proteins (Hart et al., 2007). Piraveenan et al. used topological connectivity, as well as the percolation states of individual nodes in network percolation scenarios (such as infection transmission in a social network of individuals) to quantify relative impact of nodes (Piraveenan et al., 2013). Simko and Csermely applied game centrality to design more competent interventions in cellular networks (Simko and Csermely, 2013), and Szalay and Csermely developed perturbation centrality to provide a large variety of novel options to assess signaling, drug action, environmental, and social interventions (Szalay and Csermely, 2013). Wuchty recently determined minimum dominating sets (MDSet) as optimized subsets of proteins that play a role in the control of the underlying networks by enabling remaining proteins to be reached in one step. MDSet are enriched with essential, cancer-related, and virus-targeted genes. The author also compared the MDSet proteins with hub proteins and showed a higher impact of MDSet proteins on network resilience (Wuchty, 2014).

## Fourth strategy: integration of omics data with centrality measures

Until now, we reviewed how mathematical combinations of various centralities generated from complex networks can predict essential genes (Roy, 2012). It seems that the integration of biological knowledge into topological features could create an improved centrality index to find essential nodes. Some studies have also been done in that direction; in 2010, Li et al. improved the prediction of essential proteins 20% more than closeness and subgraph centralities by construction of a weighted PPI based on the combination of logistic regression-based model and function similarity (Li et al., 2010). Li et al. in 2012 introduced and validated a new centrality measure (PeC) by integration of gene expression into the yeast PPI network. In this new method, a weighting of the PPI network was proposed based on the probability of two proteins to be co-clustered and co-expressed in a given biological scenario. PeC predicted the essential proteins significantly better than the other previously proposed 15 centrality measures: degree, betweenness, closeness, subgraph, eigenvector, information, bottle neck, density of maximum neighborhood component, local average connectivity-based method, sum of edge clustering coefficient, range-limited, L-index, leader rank, moduland, and normalized α-centralities. Above all, the enhancement of PeC over the classic centralities (betweenness, closeness, subgraph, eigenvector, and bottle neck centralities) is more than 50% for the first 500 predictions (Li et al., 2012).

Very recently, Jiang et al. in 2015 developed a network-based method named NEST (Network Essentiality Scoring Tool) that improved the performance of centrality over previous related methods. NEST predicted the essential genes according to the expression level of neighbor genes connected in protein interaction network. The results obtained by the current integration showed that the predictive power of essential protein according to this strategy is much better than the classic centralities (Jiang et al., 2015).

## Discussion

Essential genes (and their products, proteins) imply an intricate role in a cell survival and development. Topological network analyses provide opportunities for essential nodes prediction, evaluation of disease genes, and the discovery of potential drug targets (Rosamond and Allsop, 2000). Inspired by previous works in social network analysis (Freeman, 1979; Borgatti et al., 2009), it was assumed that centrality measures could predict essential nodes and several strategies have been offered to find out the relative importance of a node in complex biological networks. However, the structure of biological networks differs fundamentally from social networks especially with respect to modularity (Newman and Park, 2003). Another issue is the dynamic nature of biological entity relationships. For instance, not all relationships may exist simultaneously even in a perfectly mapped network (Han et al., 2004). Therefore, the results of centrality indices in the prediction of essential nodes were not satisfactory in various studies. One of the proposed solutions is to apply functional methods in this context according to the type of biological networks to be analyzed. Such methods integrating other aspects of biological knowledge could be very helpful. In addition, ranking genes or proteins through more biologically driven features such as physicochemical properties of bio macromolecules, intrinsic disorder property of proteins, co-expression of biological entities, gene clusters, protein complexes, protein localization, gene ontology, enrichment analysis, two-dimensional annotation of genomes, different types of promoters, and epistatic interaction will be of interest. Now that more biological data is available, it is time to improve over the pure topological measures and redefine the concept of centrality on the basis of specific properties of biological functions. A systematic look into the biological concepts is required; implying that several features could be involved and their combination would result in an improved biological centrality. More detailed analyses and discussions among researchers are needed to decide upon the parameters to be combined with different centrality measures for the prediction of essential genes in context specific biological networks. There is no particular reason to expect an exact match between network topology and biological functions. As such these tools provide the basis for “intelligent guessing.” In view of the complexity of biological networks and the difficulties to generate experimental data for other analyses, providing hints can prove already very useful.

## Author contributions

All authors listed, have made substantial, direct and intellectual contribution to the work, and approved it for publication.

### Conflict of interest statement

The authors declare that the research was conducted in the absence of any commercial or financial relationships that could be construed as a potential conflict of interest.
